# Yemurai Machirori: living with diabetes, struggling to be seen

**DOI:** 10.2471/BLT.21.030421

**Published:** 2021-04-01

## Abstract

Yemurai Machirori talks to Gary Humphreys about living with type 1 diabetes and fighting for improved access to diabetes medicines and technologies.

Q: You were diagnosed with type 1 diabetes at the age of 11. What do you remember of that experience?

A: It was a shock. My mother had taken me to the doctor for a check-up because she thought I was too skinny. Despite the fact that my mother also had the disease, she was not expecting it, and neither was I. Also, I was the last of three siblings and my siblings were both fine.

Q: Do you remember the first time you injected yourself with insulin?

A:**Vividly. It was just after my diagnosis and a nurse in the hospital showed me how it was done. She handed me the syringe and said you are going to be doing this for the rest of your life, so try it. And she stood there and watched me take my shot. It was a bit abrupt, but quite empowering really. I remember my mom coming to see me at the hospital where I had been admitted and I said, ‘Oh I’ve got to give myself my shot at the nurse’s station,’ and she looked at me like I was crazy.

Q: Did it help that there was someone in your family who knew how to manage the condition?

A: I had grown up seeing my mom take insulin and give herself pin pricks to test her blood glucose, so it was familiar to me. Also, I thought that if she can do it, I can do it too. But it was still hard being the kid with diabetes. I grew up in a community where I did not know any other child with diabetes, and the day-to-day monitoring of my blood sugar and injecting was hard. I have vivid memories of my mother waking me up at 3am to monitor my blood sugar.

Q: How often do you have to inject insulin?

A: I have to inject myself several times a day, mostly four times.

Q: How hard is it for you to get insulin and the other medical products you need?

A: It’s relatively straightforward for me. My parents both have jobs with health insurance covering the services and supplies I need, and living in Harare, those services and supplies are generally available. Overall, I have been very lucky.

Q: When did you become aware that other children were not so fortunate?

A: I was aware from an early age. I remember saying to my mother that I wanted to help other children when she first took me to the hospital. But, later on, I had experiences that really brought it home to me, especially when I started working with the Zimbabwe Diabetes Association. I had just finished my A levels (secondary education certificates) and I was helping with diabetes screening and education. It was the first time I came into contact with other people with diabetes. I remember this young boy, he was 4 or 5 years old. His mother had brought him into the city to get insulin and diagnostic test strips. The boy was quite shy, and obviously apprehensive about getting an injection. Anyway, I laughed with him a bit and I said the next time I want to see you give yourself a pin prick and take your own blood sugar. Two months later I learned that he had died. His mother had been unable to pay the bus fare to come into the city. That made a big impression on me. I also had some personal experiences that definitely influenced my thinking.

Q: Can you give an example?

A: When I went to high school, I was very aware of being “different”, maybe because of the new environment and the new people, but ironically it was someone I had known for years who made me truly feel like an outsider. This girl I had known for years came up to me and said we don’t want to see you giving yourself those scary injections anymore, so you need to go and do it in the bathroom. I did what she said and dropped a vial of insulin which broke on the floor. This was in 2008, when insulin was scarce in Harare and I was scared to say what had happened to my mom. And then I did it again! The second time I got down on my knees and drew the insulin into the needle off the bathroom floor.

“I […] drew the insulin into the needle off the bathroom floor.”

Q: You weren’t afraid of getting an infection?

A: Of course I was, but I felt like I had no choice. It was an awful moment, but it really made me want to stick up for myself and for people like me. So I went back to injecting in the classroom and when the girl came to me I said, “If you are uncomfortable look away, or go out of the room, but I am not going back to the bathroom. I am not going to make myself invisible.” It is one of the big problems we have as a group – our invisibility. That is something that a health ministry official told me once. He said you people living with type 1 diabetes are not doing enough to get noticed or be heard. He said you need to get together and make some noise if you are going to break through the wall of public indifference.

Q: Is that what you are doing in your advocacy: trying to make some noise?

A: Well, hopefully not just noise! But yes, we are trying to make our voice heard, raise awareness and educate people, notably through the social media space which is increasingly important, especially in these travel-restricted times.

Q: Can you give an example?

A: One good example would be the #insulin4all campaign which was launched in 2014 by The Pendsey Trust (a diabetes charity), and T1International, which is one of the organizations I work with. The #insulin4all campaign aims to unite the diabetes community to fight for access to diabetes supplies, care and treatment for everyone. I encourage everyone to read and share their posts!

Q: How did you make your start in diabetes advocacy?

A: I really got started with the International Diabetes Federation, an umbrella organization that brings together over 240 national diabetes associations in 168 countries. They have a Young Leaders in Diabetes Programme which they set up in 2011 to empower young people with diabetes through education and by bringing them together in events all over the world. I was chosen to be a Young Leader to represent Zimbabwe in 2013. It was a great experience and allowed me to travel to places I would never have seen otherwise, including Melbourne, Australia which is where I first connected up with T1International.

Q: What are your main issues?

A: Getting the price of insulin and other diabetes-related medical products down is a big one. As in many lower-income countries, the high cost of insulin is a major barrier to access in Zimbabwe. If I had to pay for my insulin out of pocket it would cost me around US$ 135 a month, which is roughly half what I currently earn! Luckily the cost of my insulin is covered by my parents’ insurance for most of the year. For people without medical insurance it is much worse. It is also important to remember that insulin is not the only thing you have to buy. There’s also the cost of the diagnostic strips which are used to determine your blood glucose. Our insurance used to give us a glucometer (a device used to read blood sugar) and 50 test strips per month. But two years ago, coverage stopped, and we had to buy test strips over the counter. At first that was fine, but since last year test strips have almost doubled in price, going from around US$ 15 to US$ 27 for a box of 50. I need between 50 to 150 strips per month which means I can spend up to half my monthly income just on strips. Naturally, I sometimes think maybe I will test my blood sugar less frequently, which is not the best way to approach monitoring.

“One of the big problems we have as a group [is] our invisibility.”

Q: Are insulin and other diabetes products available through the public health system?

A: Not free of charge. Government pharmacies do sell insulin at subsidized prices if they have it in stock, but that is a big “if”. So that is another big issue for us – making sure that governments prioritize procurement and supply. Quality of care is another key issue. Most of the time, people go to community clinics as their first port of call and the clinics refer them to the central hospitals in most cases, but it is not always easy for people to get to the hospitals. Of course, all of these issues have been exacerbated by the pandemic. Resources are being taken away from essential services like diabetes care and people are scared to go to clinics and hospitals because they’re afraid of catching the virus. The last big issue is the basic lack of awareness about type 1 diabetes. That’s why I am focusing so much of my effort on raising awareness. I write articles for the local newspaper and two years ago I started doing peer-to-peer consultations with children and their families at a local hospital and giving advice to doctors from a patient’s perspective. So far none of the children I have spoken to have been admitted to the hospital with diabetes-related issues, so I count that as a definite win. Going forward, and once I am done with my bachelor’s degree, I am hoping to get training in chronic disease counselling.

Q: You mentioned the impact of the pandemic on the health system in your country. What about its impact on diabetes advocacy?

A: Sometimes I feel like we are drowning in COVID! The pandemic is certainly making it harder for us to be heard, which is one of the reasons the 100-year anniversary of insulin’s discovery is such a great opportunity to get our message across.

